# Underutilisation of nuclear medicine scans at a regional hospital in Nigeria: need for implementation research

**DOI:** 10.3332/ecancer.2020.1093

**Published:** 2020-08-28

**Authors:** Akintunde T Orunmuyi, Ismaheel O Lawal, Omonefe O Omofuma, Olalekan J Taiwo, Mike M Sathekge

**Affiliations:** 1 Department of Radiation Oncology, College of Medicine, University of Ibadan, Ibadan, Nigeria; 2 Department of Nuclear Medicine, Steve Biko Academic Hospital and University of Pretoria, Pretoria, South Africa; 3 Department of Epidemiology and Biostatistics, Arnold School of Public Health, University of South Carolina, USA; 4 Department of Geography, Faculty of the Social Sciences, University of Ibadan, Ibadan, Nigeria

**Keywords:** scintigraphy, developing countries, nuclear medicine imaging, SPECT, PET, bone scan, cancer

## Abstract

**Background:**

Nuclear medicine needs better integration into the Nigerian health system. To understand the relevant public health initiatives that will be required, this study assessed the pattern of nuclear medicine imaging services at the first nuclear medicine centre in Nigeria from January 2010 to December 2018.

**Methods:**

The data of consecutive nuclear medicine (NM) scans performed between 1st January 2010 and 31st December 2018 at the NM department in a tertiary hospital in Nigeria were extracted from patient records and analysed using SAS version 9.4 (SAS Institute, Cary, NC). The National Cancer Institute’s Joinpoint software and QCIS (QGIS project) were used to estimate imaging trends and geographical spread of patients.

**Results:**

An average of 486 scans per year was performed during the study period. Patients travelled from 32 of Nigeria’s 36 states, and the majority (65%) travelled more than 100 km to obtain NM scans. Bone scans accounted for 88.1% of the studies. The remainder were renal scintigraphy (7.3%), thyroid scans (2.5%), whole-body iodine scans (1.7%) and others (0.4%).

**Conclusions:**

NM in Nigeria appears underutilised. Furthermore, the studies to characterise the access gaps and implementation needs will contribute to the design of practical strategies to strengthen NM services in Nigeria.

## Introduction

The availability of nuclear medicine in Sub-Saharan Africa has increased significantly in the past two decades [1]. Whilst South Africa has the most advanced nuclear medicine (NM) practices on the continent, spanning over six decades, the growth of NM in most of the other Sub-Saharan African countries is a recent event [1, 2]. NM diagnostic and treatment procedures are among the most powerful analytic tools for decision-making in the management of cancer, cardiovascular and neurological diseases. Appropriate utilisation of NM tools has led to fewer patients undergoing invasive and costly tests and sometimes unnecessary treatments including invasive surgeries [3]. The impact of NM on the management of cancer led to the strengthening of NM capacity by the International Atomic Energy Agency (IAEA) among member states in Africa, as a part of international cancer control efforts [1–5]. However, the growth of NM in Sub-Saharan Africa continues to face many challenges. The known barriers include ageing equipment [4], culturally embedded dread of radiation [6], logistic challenges with radionuclide delivery [3, 7, 8], lack of health insurance [9], as well as other systemic barriers that limit health systems in developing countries [2, 10].

The incidence of cancer is increasing in most of the African countries [11–14]. The demographic and epidemiological changes are leading to a rise in the non-communicable disease burden on public healthcare in Nigeria, Africa’s most populous country [15]. As a part of the national strategic health plans, NM planners in Nigeria projected that the country would require a minimum of 10 NM centres to provide equitable access to NM services in the country. Using their intuitive judgement, the sites of the ten NM centres were in tertiary hospitals that will provide radiation oncology services [8]. Having received a grant from the IAEA, the centre under review was established in 2006 as the first NM centre in Nigeria. It is geographically located in the southwest region of the country at Nigeria’s first tertiary hospital and oldest medical school [16]. The second centre was opened in 2007 at the Nation’s capital city, which is geographically in the northern central region of the country.

Both centres have a similar complement of NM physicians, which are in tertiary hospitals and major regional referral centres for radiotherapy. However, the centre under review is more established with four radiopharmacists, a dedicated medical physicist and two gamma cameras—including the only hybrid single-photon emission computer tomography/computer tomography (SPECT/CT) scanner in West Africa [17]. Furthermore, it is purpose built to accommodate two positron emission tomography (PET) scanners and a cyclotron and recently expanded from two to ten isolation rooms for radionuclide therapy, making it the largest NM facility in the country. The second centre is equipped with a double-head SPECT camera and has two radiopharmacists and two NM physicians. For unknown reasons, NM services have been interrupted for prolonged periods in the past 5 years. As a result, opportunities to scale up existing capacity, create a better understanding of the benefits of NM and invest in research and education to support its growth in Nigeria are desired. To inform the relevant public health initiatives that will be required to promote NM in Nigeria, this study assessed the pattern of NM imaging services at the first NM centre in Nigeria from January 2010 to December 2018.

## Subjects and methods

The medical records of consecutive patients who underwent NM scans at the Department of Nuclear Medicine at the University College Hospital, Ibadan, between January 2010 and December 2018 were retrospectively reviewed. The data on approximately 3,500 studies from 2006 to 2009 had been lost due to damage to the external hard drive for image storage and several missing pages on the data entry book. We collected sociodemographic and clinical information including age, sex, residential address, referral details, type of scan each patient received and the indication for the scan. A paediatric patient was defined as any patient aged 16 years or younger at the time of imaging. Furthermore, categories were created for age (missing, ≤16 years, 17–30 years, 31–40 years, 41–50 years, 51–60 years, 61–70 years or ≥71 years ), sex (male or female), type of referral hospital (in-hospital and outside hospital), type of scan, indication for referral (cancer related or non-cancer related) and referral physician speciality (unknown, surgical speciality, medical speciality, radiation oncology, paediatrics or others). The human research ethics committee of the institution approved this study, ethics number UI/EC/20/0198, and waived the need for patients consent due to the retrospective design of this study. The National Cancer Institute’s Joinpoint software was used to estimate the annual percentage change (APC) in imaging/scans from 2010 to 2018.

### Referral pattern of patients

Referral patterns were characterised by the type of referral hospital and referring physician speciality. The residential address provided by patients was used to determine the state of residence in Nigeria. Subsequently, the geospatial data were generated from the centroid coordinate of each state in Nigeria and was obtained from a digital map of Nigeria. New columns (latitude and longitude) were created, and the corresponding centroid coordinate of each patient was added. The file was saved as a text file and imported into the open-source QGIS software. The distance to the nearest hub algorithms was implemented based on the data. Subsequently, the distance travelled to obtain NM scans was estimated. A 100 km distance was empirically chosen to categorise the distance travelled to obtain scans into two groups (≤100 km or >100 km).

### Categorisation of radionuclide scans

We performed all scans in accordance with the international guidelines as published by the European Association of Nuclear Medicine and the Society of Nuclear Medicine and Molecular Imaging. Procedures were characterised according to the common single-photon emission computer tomography (SPECT) scan types (bone, renal, thyroid or whole-body iodine scans or others). Less commonly requested SPECT scans include brain, cardiac, lung and gastrointestinal (GI) scans and were grouped under the category ‘others’. Indications for NM scans were further subclassified as follows: common oncologic (cancers of the breast, prostate, cervix and GI tract), less common oncologic (all other cancers) and non-oncologic indications.

### Statistical analysis

Descriptive statistics were assessed for the means and standard deviations (SD) for continuous variables, whereas the percentages were assessed for categorical variables. All statistical analyses were conducted using SAS version 9.4 (SAS Institute, Cary, NC) and the National Cancer Institute’s Joinpoint software. A statistical significance was set at *p* < 0.05.

## Results

A review of the medical records showed that a total of 4,370 nuclear medicine scans were conducted between January 2010 and December 2018 at the University College Hospital, Ibadan. These scans were performed in 3,802 patients. A total of 568 repeat/follow-up scans were performed in 443 patients, ranging from 1 to 6 scans per patient.

### Demographic characteristics of patients

Table 1 shows the general characteristic of patients. The mean age was 53.7 ± 16.5 years (range: 0.1–93 years), and 58% were done in females. The highest proportion of nuclear medicine scans was conducted in persons aged between 51 and 60 years (23.5%). The paediatric age group accounted for 3.4% of scans performed.

### Referral patterns

**Figure 1.**
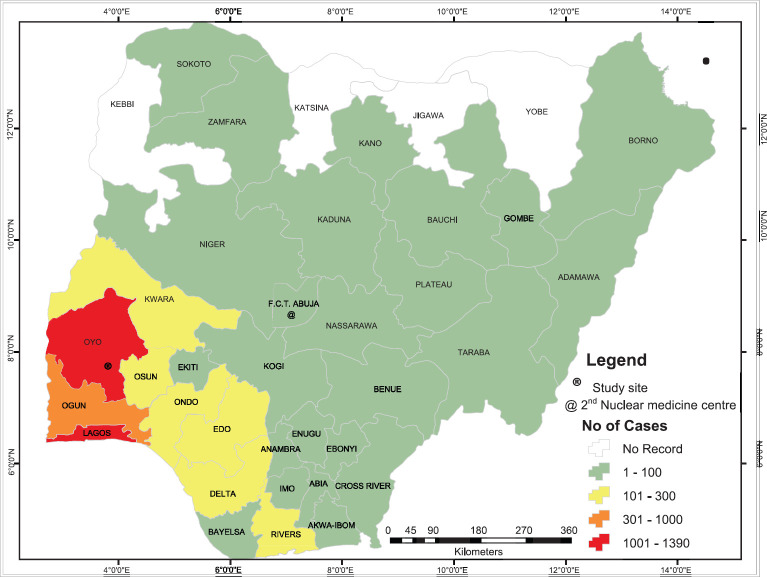
Number of cases referred for nuclear medicine scans from each state.

**Figure 2.**
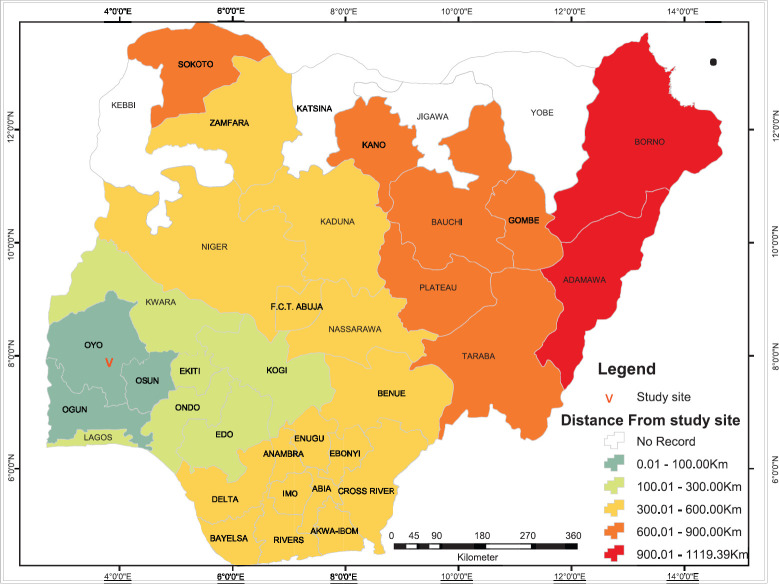
Travel distance to obtain nuclear medicine scans at study site.

**Figure 3.**
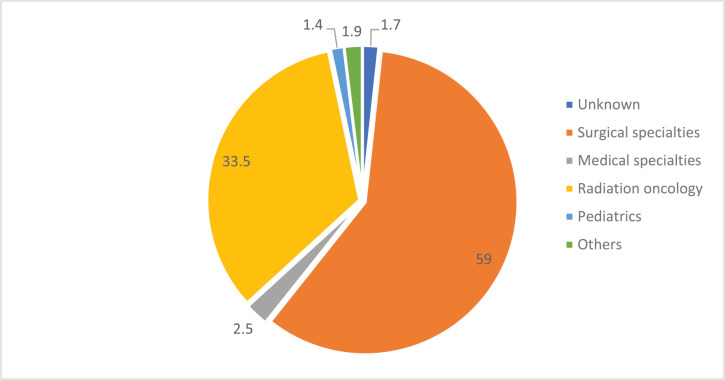
Distribution of nuclear scans by the source of referral (*n* = 4,370). *Others include family medicine, community medicine, nuclear medicine and self-referrals.

Patients travelled from all but four states in Nigeria (Figure 1), and most (65%) travelled more than 100 km to obtain scans (Figure 2). Figure 3 shows the speciality of referring physicians during the study period. Surgical specialities and radiation oncology accounted for 59% and 33.5% of total referrals, respectively.

### Characteristics of nuclear medicine scans

Bone scanning with technetium-99m (^99m^Tc)-labelled diphosphonates was the most common procedure (88.2%), followed by renal scans (7.3%), whereas pertechnetate thyroid scans and whole-body iodine (WBI) scans using radioactive iodine-131 (^131^I) accounted for 2.5% and 1.7% of scanning procedures, respectively (Table 1). Overall, 86.3% of scans performed were for oncology indications. Table 2 shows the common and less common oncology indications for scans. Female breast cancers (44.8%), prostate cancers (30%), cervical cancers (1.7%) and GI cancers (1.5%) were the top four. Among the less common oncologic indications, head and neck cancer was the most common, also accounting for 3.2 % of overall scans.

Only 585 scans were carried out for non-oncologic indications and are shown in Table 3. Renal scans (53%) were the most common non-oncologic scans, followed by bone (25.5%) and thyroid (19%) scans. Less commonly performed non-oncology scans during the study period included hepatobiliary scans using ^99m^Tc-labelled iminodiacetic acid derivatives, parathyroid scans using the cardiac imaging agent MIBI (hexakis methoxy-isobutyl-isonitrile), lymphoscintigraphy and gastric emptying scan. Technetium-labelled mercapto-acetyl-triglycine (^99m^Tc-MAG3) and technetium labelled-diethylenetriaminepentaacetic acid (^99m^Tc-DTPA) accounted for 90% of renograms, whereas the remainder were ^99m^Tc-DMSA scans (technetium-99m- labelled dimercaptosuccinic acid). Furthermore, an evaluation of non-oncologic indications for scans showed that that scans for the evaluation of pain/inflammation were the most common non-oncologic bone scan followed by scans to evaluate for infection. Notably, all requests for infection imaging were from in-hospital orthopaedic surgeons.

### Trend analysis

The trends of scans performed from 2010 to 2018 showed a significant increase in the APC of renal and thyroid scans by 21.4% and 33.6%, respectively. Conversely, bone scans decreased by 7.7%, but this was not statistically significant. Furthermore, the analysis of the trend for bone scans showed a significant decrease in the APC for breast cancer (10.9%), whereas bone scans for prostate cancer increased by 5.1%.

**Figure 4.**
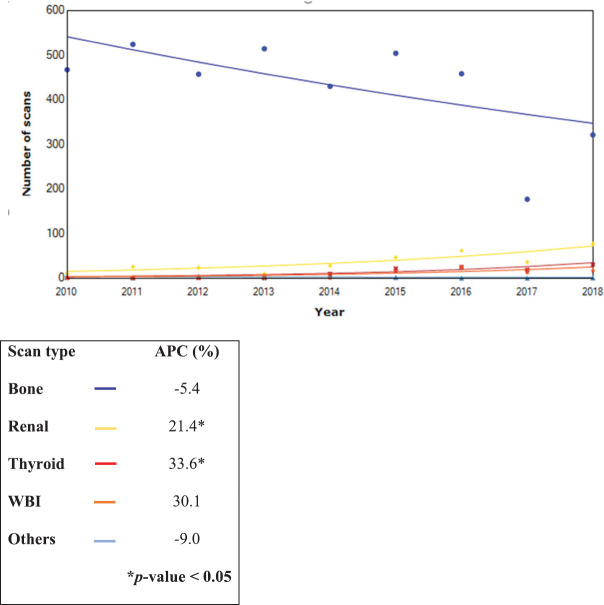
Trends in the type of nuclear scans from 2010 to 2018 (*n* = 4,370).

**Figure 5.**
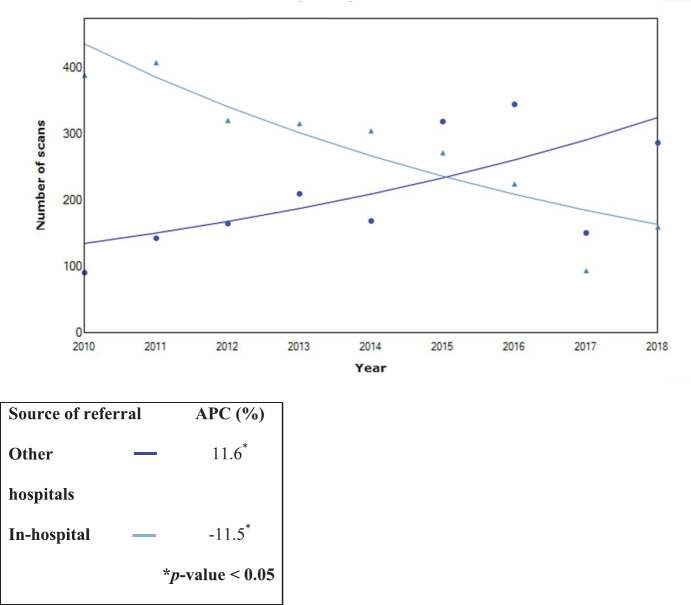
Trends in all nuclear scans from 2010 to 2018 by referral source (*n* = 4,370).

**Figure 6.**
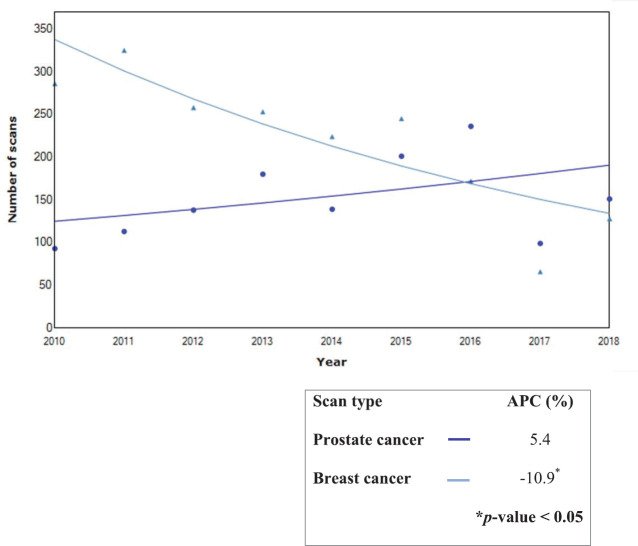
Trends in bone scan for prostate and breast cancers during 2010–2018.

Overall, a significant decline in in-hospital requests (APC: 11.6%) and an increase in outside hospital requests (APC: 11.60) were noted (*p* < 0.05). The trend data are shown in Figures 4–6.

## Discussion

This is the first study reporting on NM utilisation and referral patterns in Nigeria, the most populous African nation [15]. The volume of scans averaging 486 scans per annum is consistent with the other studies reporting similar low throughput of nuclear medicine departments in Sub-Saharan Africa [2, 4]. Besides the specific barriers of NM in Nigeria [4, 6–9], general challenges that affect oncology and healthcare delivery significantly impact on NM scans. Since the majority of the referrals come from oncologists, radiotherapy equipment downtime and industrial strike actions by health professionals trade unions impact on NM services [18]. External strikes (aviation and logistics) are not only less frequent but also halt NM service delivery. For instance, since the global COVID-19 pandemic started, an import of radionuclides from overseas has been disrupted. The affordability of scans also plays a major role in the utilisation of NM procedures in Nigeria [9]. In our experience, camera downtime as a factor for under utility of NM is infrequent. Its effects are minimised due to the availability of two gamma cameras.

The low utility of NM scans could be partly explained by the accessibility of services. With only two centres nationwide, patients would have to travel long distances to obtain services [9]. Although patients travelled from all but four states in the country, the numbers of scans diminished with distance travelled (Figure 1). The four states without referrals are a part of six states in Nigeria with remarkably high rates of severe malnutrition [19]. Therefore, it is likely that travel distance and socioeconomic factors play a role in the utility of NM services. This could be evaluated in the further studies.

The results show that few NM scans were performed for non-oncologic indications. Remarkably, no cardiac imaging was conducted during the period under review. The absence of nuclear cardiology imaging may have contributed to the low scan volumes in this study. Nuclear cardiology, in general, notably stress myocardial perfusion using single-photon emission computed tomography (SPECT), is underutilised in many developing countries [20–22]. Myocardial ischaemia appears to be an infrequent cause of hospital deaths in Nigeria despite population-level changes in cardiovascular disease mortality and morbidity [23–25]. The ability to foster the adoption and expansion of nuclear cardiology in developing countries may be blunted by the marked decline of SPECT cardiology imaging globally [26].

Infectious diseases continue to be the leading cause of mortality in Sub-Saharan Africa [11]. This proposes that the need for infection imaging will be high. The utility of NM for infection imaging was low and limited to referrals from in-hospital orthopaedic surgeons. Although in-house orthopaedic surgeons are renown experts in the country and more informed about NM services [27], we do not believe that they are utilising NM scans to the maximum possible. Feedback of their dissatisfaction with bone scans for the evaluation of prosthetic joint inspired efforts to introduce technetium-labelled ubiquicidin peptide (99mTc-UBI). However, the local research studies to evaluate its impact are required. Conducting research has been shown to facilitate the adoption of new techniques in small-scale initiatives [28]. Given the recent development of infection-specific radiopharmaceuticals such as 99mTc-UBI [29], advances in SPECT quantification and standardisation of imaging procedures [30], the approaches to raise awareness about NM infection imaging and its potential adoption are needed.

Overall, the low utility of NM for non-oncologic scans may not be unrelated to the promotion of NM at its founding as an important tool for cancer management [4, 5]. Furthermore, the studies to explore other unknown factors that play a role in the low throughput of NM services in Nigeria and other Sub-Saharan African countries are warranted. Implementation science has been proposed as a means to foster the adoption of evidence-based strategies for health in low- and middle-income countries [28, 31]. Investigating the challenges of implementation and adoption of NM in sub-Sahara Africa should not be overlooked.

The predominant use of diuresis renography among adults in this study is noteworthy. It contrasts with the reports from developed countries, where diuresis renography is predominantly used in children for the early detection and management of congenital abnormalities of the kidneys and urinary tracts (CAKUT) [32–35]. In Nigeria, CAKUT is responsible for between 7.8% and 17.5% of paediatric admissions and a major cause of chronic kidney disease among children [32, 36–38]. Coordinated efforts to improve the diagnosis and treatment of renal diseases in childhood are needed and may impact on renal diseases observed in adulthood. Opportunities for multidisciplinary research and coordination of public health paediatric care in proximity to NM centres should be explored further.

The role of NM in thyroid disease continues to be of interest. The current role of thyroid scintigraphy for diagnosis is adjunctive [39]. Several radionuclides are used for thyroid imaging, where NM contributes to the treatment of both oncologic and non-oncologic conditions. Radioactive iodine (123I) and pertechnetate (99mTcO4-) are the radionuclides used for imaging, whereas 131I is used for treatment. Similar to the other reports, this study shows 99mTc-pertechnetate as the main isotope used for thyroid imaging in resource-poor settings. Its major indication for use is to evaluate clinically confirmed Graves’ disease. Other indications include evaluation of thyroid nodules and thyroiditis to locate ectopic thyroid tissue [39, 40]. Its low cost, ready availability, rapid imaging and lower absorbed dose are major advantages. For patients undergoing radioactive iodine therapy for benign and overactive goitres, it is routinely used to guide empirical dosing [6, 41]. However, personalised radioiodine therapy is best achieved by a radioiodine uptake test [39].

Radioactive iodine 131I therapy (RAIT) is the first targeted theragnostic radionuclide in NM and plays an important role in thyroid carcinoma treatment [42]. The reports of the use of RAIT in Nigeria predate the establishment of the NM imaging facilities [43–45]. The rising patterns of thyroid and whole-body scintigraphy with 131I (WBI) in this study provide recognition for the impact of NM in Nigeria. WBI is recommended for staging of patients with suspected metastases from differentiated thyroid carcinomas. The use of the same radionuclide (131I) for diagnosis and treatment has its controversies [46]. However, it remains useful for identifying patients who will benefit from 131I from the other forms of therapy in the case of poorly differentiated or dedifferentiated thyroid carcinoma [42, 46]. WBI provides information on nodal and distant metastases for staging of the disease and gives a visual representation for monitoring treatment response and detection of recurrence during follow-up [42, 46]. Therefore, the routine administration of radioactive iodine 131I without radionuclide imaging is not recommended [40, 42, 46]. Opportunities to educate oncologists/endocrinologist on the role of imaging for radioactive iodine therapy must be sustained.

Challenges with radionuclide supply continue to impact on timely access to NM services and its growth in Nigeria [8]. This may contribute to the observed stable imaging trends despite population health evidence shows the need for NM. The decline in bone scan for breast cancer may indicate the impact of evidence-based research on clinical practice. In a prior study, we had shown that bone scan was frequently showed metastases in patients with stage III and IV disease. Hence, the routine use of bone scan for staging is only in these patients [47]. Consistent with global patterns, bone imaging with technetium-labelled diphosphonates was the most common procedure in the centre. It is well established for staging of patients with prostate, breast, small-cell lung tumours and other cancers which frequently metastasise to bone [48, 49]. The top four oncologic indications for NM scans in this study were consistent with country figures [7, 23].

General advances in imaging are increasingly revealing the limitations of bone scanning, particularly for modifying treatment outcomes and early detection of treatment response in cancers [50–52]. Recent PET tracers are promising for the application of PET in the initial evaluation of several oncologic diseases [53, 54]. Since automated synthesis systems have increased the reliability, reproducibility and safety of radiopharmaceutical productions [55–57], the absence of PET services in Nigeria is largely due to the high cost of investment. In the southern hemisphere of Africa, the availability of PET is limited to South Africa and, recently, Kenya. Recent advances in SPECT technology are projected to advance SPECT closer to PET imaging [58]. However, it will require matching advances in SPECT radiopharmacy and affordability within the reach of low-resource countries. Furthermore, the feasibility analysis on the need and utilisation of technological advances in NM technologies (Cyclotron, PET, SPECT) in Africa are warranted in the future.

## Conclusion

Nuclear medicine in Nigeria has been sustained for 13 years but appears underexplored and underutilised. However, the limited availability of NM services creates unequal access for patients who require these services. Differential and contextual factors contribute to the slow growth of NM in Nigeria and increase the complexity of interventions that are required to boost it. Furthermore, the studies to characterise the access gaps and implementation needs are desired. Pragmatic steps to strengthen NM services in Nigeria are critical to appreciate its potential beyond the current level of utilisation in the Nigerian health system.

## List of abbreviations

NM: Nuclear medicine
NCD: Non-communicable diseases
IDA: Iminodiacetic acid
GI: Gastrointestinal
WBI: Whole-body iodine
^131^I: Radioactive iodine 131
^99m^Tc-MAG3: Technetium-99m-labelled mercapto-acetyl-triglycine
^99m^Tc-DTPA: Technetium-99m-labelled-diethylenetriaminepentaacetic acid
^99m^Tc-DMSA scans: Technetium-99m-labelled-dimercaptosuccinic acid
MIBI: (Hexakis) methoxy-isobutyl-isonitrile
APC: Annual percentage change
SPECT: Single-photon emission computed tomography
CAKUT: Congenital anomalies of the kidneys and urinary tracts
PET: Positron emission tomography
RAIT: Radioactive iodine I-131 therapy

## Funding declaration

No funding was received for this work.

## Declaration of conflicts of interests

The authors declare no conflict of interest.

## Figures and Tables

**Table 1.** Baseline characteristics

Characteristics	
Age, years, range (mean ± SD)	0.1–93 (53.7 ± 16.5)
Age group, *n* (%)	
Missing ≤16 years 17–30 years 31–40 years 41–50 years 51–60 years 61–70 years ≥71 years	2 (0.1) 147 (3.4) 188 (4.3) 498 (11.4) 872 (20.0) 1,028 (23.5) 1,007 (23.0) 628 (14.4)
Sex, *n* (%)	
Male Female	1,842 (42.2) 2,528 (57.9)
Travel distance, *n* (%)	
≤100 km >100 km	1,542 (35.3) 2,828 (64.7)
Hospital referral	
In-hospital Outside hospital Unknown	2,490 1,878 2
Type of scan, *n* (%)	
Bone^a^ Renal Thyroid Whole-body iodine Others	3,852 (88.2) 320 (7.3) 109 (2.5) 72 (1.7) 17 (0.4)
Overall indication for scans, *n* (%)	
Common oncologic indications Less common oncologic indications Non-oncologic indications	3,446 (78.8) 339 (7.8) 585 (13.4)

a Including three Tc99m-UBI and two Tc99m- PSMA scans.

**Table 2.** Oncologic indications for nuclear medicine scans during the study period.

Common indications, *n* (%)		Less common indications, *n* (%)	
Breast cancer	1,957 (44.8)	Head and neck cancers^a^	138 (3.2)
		Connective tissue cancers	48 (1.1)
Prostate cancer	1,350 (30.9)	Urogenital cancers	39 (0.9)
Cervical cancer	75 (1.7)	Cancer of unknown origin	38 (0.9)
GI cancer	64 (1.46)	Haematologic cancers	29 (0.7)
		Lung cancers	23 (0.5)
		Others^b^	24 (0.5)

a Includes whole-body iodine scans and thyroid scans for thyroid cancer

b Brain cancers, mediastinal tumour, gynaecological cancers (uterine, vaginal and vulva), lymphoedema

**Table 3.** Non-oncologic nuclear medicine scans during the study period.

Description	Age ≤ 16 years	Age > 16 years
Renal scans		
Diuretic renograms (MAG3, DTPA)	103	178
DMSA	9	21
Bone scans		
Pain/inflammation	47	55
Infection	28	11
Fracture	1	7
Thyroid scans		
Hyperthyroidism	1	96
Other thyroid conditions^a^	1	11
Other scans		
Hepatobiliary scans	11	-
MIBI Parathyroid	-	3
Lymphoscintigraphy	1	-
Gastric emptying	-	1
Total	202	383

a Lingual thyroid, multinodular goitre, cold nodule evaluation
